# A Rare Neurological Complication of Ranolazine

**DOI:** 10.1155/2013/451206

**Published:** 2013-07-01

**Authors:** Jahan Porhomayon, Gino Zadeii, Alireza Yarahmadi

**Affiliations:** ^1^VA Western New York Healthcare System, Division of Critical Care and Pain Medicine, Department of Anesthesiology, School of Medicine and Biomedical Sciences, State University of New York at Buffalo, Buffalo, NY 14215, USA; ^2^University of Iowa, Mason City Cardiology, Mason City, IA 50401, USA; ^3^Director of Mercy North-Iowa Neurology and Sleep Laboratory, University of Iowa, Mason City Neurology, Mason City, IA 50401, USA

## Abstract

Myoclonus is not a known side effect of ranolazine. We report a case of myoclonus in a 72-year-old female who underwent cardiac catheterization for angina and was started on ranolazine after the procedure. Two days after ranolazine therapy on 1000 mg per day in divided doses, myoclonus developed, which severely impaired her normal activity. Her symptoms resolved 2 days after discontinuation of ranolazine. Ranolazine was resumed after discharge from hospital with recurrent myoclonus after two days of therapy. The causal relationship between ranolazine and myoclonus was suggested by cessation of myoclonus after ranolazine was discontinued.

## 1. Introduction

Chronic angina is a debilitating condition affecting nearly 6 million Americans. Current standard therapy includes beta-blockers, calcium channel blockers, and long acting nitrates. Some patients may be intolerable to standard therapy due to their side effects [1]. Ranolazine is new agent introduced into clinical practice in 2006. It is an extended release antianginal drug and is intended to act without reducing heart rate or blood pressure. Ranolazine is specifically indicated for the treatment of chronic angina in patients that failed previous anti-ischemic therapy [2]. It is contraindicated in patients with QT prolongation [3]. It has a piperazine compound that belongs to a group known as partial fatty-acid oxidation inhibitors [4]. Initially, the main anti-anginal effects of ranolazine were thought to be related to the actions of ranolazine to shift adenosine triphosphate (ATP) production away from fatty-acid oxidation toward glycolysis [5, 6]. Recent evidence suggests that ranolazine is an inhibitor of the late sodium current which results in a reduction of the intracellular sodium and calcium overload in ischemic cardiac myocytes [7–9].

## 2. Case Report

 This is a 72-year-old female who presented to the emergency department with history of chest pain and non-ST-segment elevation myocardial infarction (NSTEMI). Her past medical history was significant for intermittent chest pain. She underwent cardiac catheterization with placement of 2 drug eluding stents and was started on ranolazine for symptomatic relief of NSTEM with angina.

Her medication list included atorvastatin 20 mg daily, clopidogrel 75 mg daily, aspirin 162 mg daily, diltiazam 60 mg four times a day, and ranolazine 500 mg twice daily. She presented 2 days after discharge with myoclonic jerks in her upper and lower extremities. She was readmitted in the hospital for evaluation of myoclonus. At the time of her hospitalization, ranolazine was discontinued and she did not have any further myoclonus. Brain MRI (magnetic resonance imaging) and blood works including liver enzymes, renal function, and electrolytes all were within normal limits. She was discharged home, and ranolazine was resumed as part of her discharge medication list. She had another episode of generalized myoclonus involving face, arms, and legs that started on the second day of her discharge. Myoclonic jerks progressively got worse; therefore, she presented to emergency room. After readmission and discontinuing ranolazine, her myoclonic jerks disappeared again.

## 3. Discussion

 Current studies evaluating the safety and side effects of ranolazine alone or in combination with other agents have not revealed myoclonus as a known side effect [10–13]. Ranolazine is usually well tolerated, and the most common adverse effects include dizziness, constipation, nausea, asthenia, syncope, headache, and abdominal pain. Ranolazine is a relatively new drug, released in early 2006, and the total experience with it is relatively limited. 

In the monotherapy assessment of ranolazine in stable angina (MARISA) trial [14], 191 patients were randomized to 500 mg, 1000 mg and 1500 mg of ranolazine, and most adverse events occurred in the 1500 mg dose range. In the combination assessment of ranolazine in stable angina (CARISA) trial [14], five cases of syncope were reported when 1000 mg twice daily dose was used; all cases involved patients on concurrent medications known to raise ranolazine plasma concentrations. However, there were no reported cases of syncope in the efficacy of ranolazine in chronic angina (ERICA) [15] trial. In the ranolazine open label experience (ROLE) trial [16], 746 patients were followed up to almost 3 years with 72 patients discontinuing ranolazine due to dizziness (11.8%) and constipation (10.9%). None of the trials mentioned above reported myoclonus as a side effect.

Ranolazine is extensively metabolized by CYP3A enzymes and, to a lesser extent, by CYP2D [10]. Due to its principal CYP3A-mediated metabolism, multiple drug-drug interactions are seen with ranolazine. Moderate to potent inhibitors of the CYP3A4 enzyme such as ketoconazole, diltiazem, verapamil, macrolide antibiotics, and protease inhibitors can increase plasma ranolazine concentrations by 2.0- to 4.5-fold. Additionally, because ranolazine also blocks persistent sodium (Na) channels both in cardiac and neuronal channels [17], it has been investigated as a promising agent for treatment of conditions resulting from neuronal excitation. Although ranolazine mainly targets persistent Na channels, it can interact with broad spectrum of Na and central nervous system channels. We believe that myoclonic reaction may have occurred as a result of ranolazine interaction with other Na channels as well as persistent resurgent sodium currents [18] leading to increased neural sensitivity. Our current understanding and knowledge regarding Na channels properties are increasing but are currently incomplete. More research is needed to improve selective targeting of Na channels in order to limit side effects of newer agents.
